# A single cut to pyroptosis

**DOI:** 10.18632/oncotarget.6142

**Published:** 2015-10-16

**Authors:** Yoon Lim, Sharad Kumar

**Affiliations:** Centre for Cancer Biology, University of South Australia, Adelaide, SA, Australia

**Keywords:** cell death, pyroptosis, caspases

Pyroptosis is a form of inflammatory cell death mediated by inflammatory caspases. Two independent studies have recently demonstrated that inflammatory caspases cleave gasdermin D and the resulting N-terminal fragment of this protein mediates pyroptosis. Here we summarize the key findings reported in these important publications.

**Figure 1:**
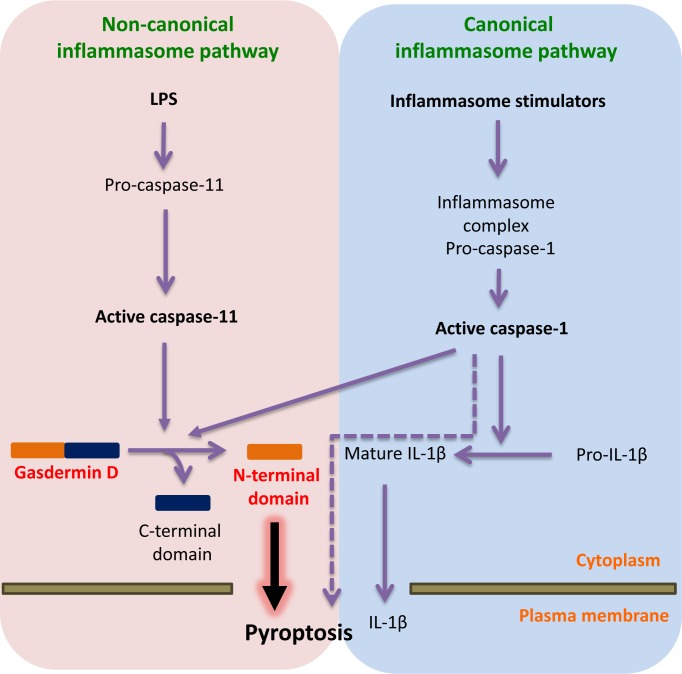
Active caspase-11 and caspase-1 cleave GSDMD to execute pyroptosis In the non-canonical pathway, LPS in the cytosol of bacterially infected cells binds to pro-caspase-11, which leads to caspase-11 activation. Shi [4] and Kayagaki [5] demonstrate that active caspase-11 processes gasdermin D (GSDMD) to release its N-terminal domain from its inhibitory C-terminal domain. The released N-terminal domain induces pyroptosis through plasma membrane breakdown and release of inflammatory contents. In the canonical inflammasome pathway, microbial pathogens and inflammatory substances are detected by various cytosolic sensor proteins which lead to caspase-1 activation through an inflammasome complex. Active caspase-1 converts pro-IL-1β to mature IL-1β which is released from the cells undergoing pyroptosis. Both studies demonstrate that caspase-1 also processes GSDMD to induce pyroptosis, however the evidence also suggests that caspase-1 may mediate pyroptosis in a GSDMD-independent manner, possibly through the cleavage of other substrates. LPS: lipopolysaccharide; IL-1β: interleukin-1 beta.

Caspases are cysteine proteases that function in apoptosis and other forms of cell deaths, such as pyroptosis, a form of proinflammatory programmed necrosis [1]. Pyroptosis is a critical innate immune defence system against microbial pathogens and features pore formation in the plasma membrane and its subsequent rupture, resulting in the release of cytosolic inflammatory contents. Activation of the inflammatory caspases caspase-1 and caspase-11, of which caspase-4 and caspase-5 are the human homologs, play a pivotal role in the execution and regulation of pyroptosis (Figure 1). In the canonical inflammasome (a multi-protein complex) pathway, caspase-1 is activated by inflammasome formation upon proinflammatory stimuli, such as microbial infection, and processes proinflammatory cytokines such as IL-1β and IL-18, triggering pyroptosis [1]. In contrast to the canonical inflammasome pathway, direct binding of caspase-11 to cytoplasmic lipopolysaccharide (LPS), a strong innate immune system stimulator, leads to non-canonical inflammasome activation to induce pyroptosis [2, 3]. While the mechanisms of inflammasome assembly are well understood, until recently, the mechanisms of how inflammatory caspases bring about pyroptosis have been unclear. Two papers recently published in *Nature* [4, 5] now describe that caspase-1 and caspase-11 mediated cleavage of a previously unsuspected culprit gasdermin D (GSDMD), is a critical step in the execution of pyroptosis.

The two groups used different strategies to arrive at their findings. Shi *et al.* [4] performed a genome-wide screen using CRISPR/Cas9 to identify essential factors in caspase-1 and caspase-11-induced pyroptosis in immortalized bone marrow-derived macrophages. Kayagaki *et al.* [5] conducted a genetic screen in mice randomly mutated by ethyl-N-nitrosourea to identify down-stream molecules of capase-11-mediated non-canonical inflammasome signalling. Both groups identified the *Gsdmd* that encodes GSDMD a protein of unknown function. The genetic screening results were confirmed through several experiments in mouse and human cells, showing that GSDMD is an essential molecule for non-canonical inflammasome-mediated pyroptosis (Figure 1). Kayagaki *et al.* also demonstrate that LPS-induced lethality is dramatically reduced in both *Gsdmd^−/−^* and *Casp11^−/−^* mice, which is critical *in vivo* data supporting that GSDMD is indeed a key substrate of caspase-11 in pyroptosis.

While data from both groups show that GSDMD is necessary for caspase-4, caspase-5 or caspse-11-induced pyroptosis, their experimental results differ on the exact requirement of GSDMD in caspse-1- induced canonical pyroptosis (Figure 1). Shi *et al.* generated *Gsdmd^−/−^* macrophages to confirm that GSDMD is essential in caspase-1-induced pyroptosis and further demonstrated that pyroptosis is prohibited upon exposure to canonical inflammasome pathway activators for a short incubation. Kayagaki *et al.* also showed similar results for a short incubation, however extended incubation (8-16 hours) did not show pyroptotic differences between wild type and *Gsdmd^−/−^* macrophages. This result suggests that GSDMD may not be a critical molecule required to mediate caspase-1-induced canonical pyroptosis. Therefore, other essential caspase-1 substrates capable of inducing caspase-1-induced canonical pyroptosis may yet to be discovered.

Shi *et al.* [4] demonstrated that after canonical inflammasome activation caspase-1 activation and IL-1β cleavage are not affected in *Gsdmd^−/−^* mouse macrophages, but the extracellular release of mature IL-1β is completely abolished. This strongly supports the argument that pyroptosis is required for IL-1β release and also suggests that GSDMD acts as downstream target of inflammatory caspases.

Utilising biochemical analyses, both groups determined that GSDMD is cleaved by caspase-1, -4/-5, and/or caspase-11 at D276 in a linker localising between the N-terminal and C-terminal domains of GSDMD. Mutagenesis on this cleavage site failed to rescue pyroptosis in *Gsdmd^−/−^* macrophages, suggesting that GSDMD cleavage is essential for pyroptosis. Both groups found that the N-terminal domain after cleavage is sufficient to induce cell pyroptosis and that the C-terminal domain inhibits this pyroptotic cell death by binding to the N-terminal domain of GSDMD. Shi *et al.* further demonstrated that the engineered GSDMD having the cleavage site of caspase-3/7 between the N-and C-terminal domains presents cell pyroptosis. These findings confirm that the activity of GSDMD is kept in check by its C-terminal domain and the N-terminal domain released by caspase-dependent cleavage induces cell pyroptosis. Further studies are required to decipher how the N-terminal domain of GSDMD mediates its cytotoxic effect.

GSDMD is a member of the poorly characterised GSDM family of proteins. Both studies were able to highlight the possible roles of other GSDM family members in pyroptosis. Genetic mutations in mouse GSDMA3 lead to hair-loss and hyperkeratosis due to chronic skin inflammation [6]. Shi *et al.* found that these mutations cause abnormal pyroptosis and most GSDMs can trigger pyroptosis with their N-terminal domains. Interestingly, GSDMD is the only member in the GSDM family cleaved by inflammatory caspases. The physiological functions of other GSDMs in executing pyroptosis following microbial infections need further investigations.

Although how exactly GSDMD cleavage product leads to pyroptosis remains to be worked out, these two studies have highlighted how the cleavage of single key substrate like GSDMD by inflammatory caspases leads to pyroptosis. This is unlike apoptosis where hundreds of substrates are cleaved by caspase-3 and several are predicted to be critical for the pursuit of dismantling the cell undergoing apoptotic death. The critical insight into the mechanisms of pyroptosis will no doubt lead to new approaches to target inflammasome-associated disorders [7].
